# Pneumococcal meningitis and vaccine effects in the era of conjugate vaccination: results of 20 years of nationwide surveillance in Germany

**DOI:** 10.1186/s12879-015-0787-1

**Published:** 2015-02-14

**Authors:** Matthias Imöhl, Jens Möller, Ralf René Reinert, Stephanie Perniciaro, Mark van der Linden, Orhan Aktas

**Affiliations:** Institute of Medical Microbiology and National Reference Center for Streptococci, University Hospital (RWTH), Pauwelsstr. 30, Aachen, Germany; Department of Neurology, Medical Faculty, Heinrich-Heine-University, Düsseldorf, Germany

**Keywords:** *Streptococcus pneumoniae*, Meningitis, Serotypes, Vaccine coverage, Antibiotic susceptibility, Age

## Abstract

**Background:**

Long-term complications and a case mortality rate of 7.5% make meningitis caused by *Streptococcus pneumoniae* a serious clinical threat. In 2006, a general pneumococcal conjugate vaccination (PCV) recommendation was issued for all children under 2 years in Germany. Here, we investigate serotype changes in meningitis cases after this vaccine recommendation.

**Methods:**

The German National Reference Center for Streptococci (NRCS) has conducted surveillance for invasive pneumococcal disease (IPD) in Germany since 1992. Pneumococcal isolates were serotyped by the Neufeld’s Quellung reaction and antibiotic susceptibility was tested using the broth microdilution method.

**Results:**

Of 22,204 IPD isolates sent to the NRCS from July 1992 to June 2013, 3,086 were meningitis cases. Microbiological and statistical investigations were performed to characterize and quantify all meningitis cases, focusing on changes reflecting implementation of the national PCV recommendation. 1,766 isolates (57.2% of meningitis cases) were from adults (≥16 years) and 1,320 isolates (42.8%) originated from children (<16 years). Overall, the leading serotypes were 14 (9.7%), 7F (7.8%), 3 (6.9%), 19F (5.7%) and 23F (5.0%). Among children, serotypes 14 (16.2%), 7F (8.9%) and 19F (7.1%) were most common, whereas among adults, serotypes 3 (9.6%), 7F (6.9%), 22F (5.0%), 23F (4.9%) and 14 (4.8%) were most prevalent. After the introduction of general PCV7/10/13 vaccination a significant decrease for most vaccine serotypes was observed. Generally, the differences in antibiotic nonsusceptibility between children <16 years and adults ≥16 were low. For macrolides in the pre-PCV7 period, a significantly higher proportion of resistant isolates was found in children (25.1%), compared to the post-vaccination period (9.7%; p<0.0001).

**Conclusions:**

Implementation of the pneumococcal conjugate vaccines broadly reduced vaccine-type meningitis cases. Changes in serotype prevalence must be continuously monitored to observe future trends concerning pneumococcal meningitis.

## Background

*Streptococcus pneumoniae* is among the most important pathogens in bacterial meningitis worldwide [1]. Meningitis occurs in all age groups, with the peak of incidence during the first two years of life [2,3], especially during months 2–12 [3-5]. Case fatality rates in pneumococcal meningitis are high, ranging from 10% up to 60%, and are higher in adults than in children [6]. Among children in Germany, mortality rates of 7.5% have been reported [7]. Despite adequate antibiotic treatment, about half of the survivors show substantial long term morbidity, as reflected by persisting neurological deficits comprising cognitive impairment, seizures, hearing loss, and other handicaps [8]. Early clinical complications, such as life-threatening increase of intracranial pressure, and late disease consequences in survivors, who frequently suffer from sustained neuropsychological impairment, contribute to the unfavorable outcome. However, the precise mechanisms of neurotoxicity are still largely unknown. Clearly, the capsular polysaccharide of *S. pneumoniae* contributes to pathogenicity [9], and pneumococcal toxins (such as pore-forming pneumolysin or hydrogen peroxide) as well as excessive host immune responses have been suggested as harmful influencing factors [10,11]. Indeed, recent studies have suggested the use of corticosteroids to limit excessive inflammatory reactions within the CNS [12]. However, regarding *Streptococcus pneumoniae*, regional epidemiologic features may affect the possible therapeutic benefit of corticosteroids [13], emphasizing the relevance of a systematic description of the respective isolates.

In Germany, a general recommendation for pneumococcal conjugate vaccination for all children under two years of age was issued in July 2006. Initially, vaccination was performed with Prevenar® (PCV7). Beginning in April 2009, with licensing of higher-valent vaccine formulations, Synflorix® (PCV10) was used, and Prevenar 13® (PCV13), which replaced PCV7, was introduced in December 2009. All vaccination costs are fully reimbursed by health insurance companies and the choice of the vaccine lies with the parents/pediatrician. Uptake of the PCV7 vaccine was high and reached 80% one year after the vaccination recommendation. Uptake of PCV10 has been low, reaching 20% of total doses only in 2009, and decreasing afterwards. Current childhood vaccine uptake lies above 90%, of which 96% is PCV13. The 23-valent pneumococcal polysaccharide vaccine (Pneumovax 23®) is recommended for adults 60 years and older, and for at-risk patients ≥2 years of age.

Several studies have reported frequencies of pneumococcal serotypes and pneumococcal vaccine coverage in invasive pneumococcal disease (IPD), but little information is available on serotype distribution among meningitis cases from Germany. Experience from other European countries as reflected by the 2011 report of the ECDC as well as by separate studies from Spain [14], England and Wales [15,16], and The Netherlands [17,18] suggest a profound impact of PCV7 vaccination programs on the incidence of pneumococcal meningitis.

Moreover, in light of the emergence of antibiotic-resistant strains, the choice of first-line treatment represents an important epidemiological issue. Finally, the possible impact of the general recommendation of pneumococcal conjugate vaccination for children <2 years, introduced in Germany in July 2006, has not yet been investigated.

Here we analyzed serotype distribution, theoretical serotype coverage of pneumococcal vaccines, and antimicrobial susceptibility of all meningitis isolates that were sent to the German National Reference Center for Streptococci (NRCS) from July 1, 1992 to June 30, 2013.

## Methods

### Study design

The NRCS has conducted surveillance for IPD in Germany since 1992. In this study, a population- and laboratory-based approach was used to collect data about meningitis among children (<16 years) and adults (≥16 years) caused by *Streptococcus pneumoniae*. Isolates were sent to the NRCS by diagnostic microbiological laboratories throughout Germany on a voluntary basis [19].

Invasive pneumococcal disease cases were defined as *S. pneumoniae* isolates from blood, cerebrospinal fluid (CSF) or any other normally sterile body fluid. Meningitis was primarily defined by the isolation of *S. pneumoniae* from cerebrospinal fluid (CSF). However, when meningitis was specified as the definite clinical diagnosis on the case form, and the samples originated from blood, these samples were also considered to represent meningitis cases, even if CSF from these patients was not received.

Microbiological diagnostic laboratories from all over Germany were requested to send isolates of invasive pneumococcal disease to the NRCS. Currently, over 300 laboratories participate, including the large, nationally-operating commercial labs. Over the years, the surveillance system has been improved. In 2001, surveillance for adults was enhanced in North Rhine-Westphalia (22% of German population), as well as in Bavaria and Saxony in 2006. On each occasion, all laboratories in the respective federal states were approached and asked to send in isolates. In 2007, a web-based surveillance system (Pneumoweb) was set up in collaboration with the Robert Koch Institute. Pneumoweb enables the laboratories to report a case of IPD via an online reporting system, and at the same time, print the reported information to accompany the IPD isolate, which can then be sent to the NRCS. The web-based system resulted in a large increase in reported cases for adults, whereas the amount of cases for children remained at the same high level.

Cases were grouped per pneumococcal season (from July to June of consecutive years) because of known infection clusters during winter.

### Microbiological investigations

Isolates were identified using standard procedures including bile solubility and optochin sensitivity. Pneumococcal isolates were serotyped by the Neufeld’s Quellung reaction using type- and factor-specific antisera (Statens Serum Institut, Copenhagen, Denmark). Minimal inhibitory concentrations (MIC) testing was performed using the broth microdilution method as recommended by the Clinical and Laboratory Standards Institute (CLSI) [20] and current CLSI interpretative criteria were used to define antimicrobial resistance. Macrolide resistance was investigated using erythromycin or clarithromycin. To assess the development of penicillin (≤0.06 mg/l, − , ≥0.12 mg/l) and cefotaxime (≤0.5 mg/l, 1 mg/l, ≥2 mg/l) resistance, parenteral meningitis breakpoints were used. *Streptococcus pneumoniae* ATCC 49619 was used as a control strain.

### Statistical methods

Statistical calculations were performed using R (version 3.0.1, 2013) and the Analysis ToolPak in MS Excel. Fisher’s exact test was used to compare differences in proportion and two-sample t-tests were used to compare differences in percent distributions. All p-values reported are two-sided. Adjustments for multiple comparisons were performed using the Dunn-Šidák correction, and the resulting β values, which determine the significance threshold, are included in the table legends. The season of vaccine introduction, 2006–07, was considered a transition period and taken out of consideration in the analyses.

### Ethics and consent

An ethical approval or patients’ consent was not required since the study only includes microbiological samples sent to the German National Reference Center for Streptococci on an anonymized basis by the sending microbiological laboratories, and did not involve human subjects or material.

## Results

A total of 3,086 isolates from meningitis cases were collected, with 1,766 isolates (57.2%) from adults and 1,320 isolates (42.8%) from children. The mean age was 34.0 years (median 37 years; range 0–99 years). More isolates were obtained from male patients (55.0%) than from female patients (43.6%). For 1.4% of isolates no information on gender was available. The mean age among children was 2.8 years (median 1 year) and among adults 57.2 years (median 59 years). The isolates were obtained from CSF (2565) and blood (521). The absolute number of meningitis cases reported to NRCS per pneumococcal season varied between 8 and 121 (mean 62.9; median 66) for children, and between 26 and 183 (mean 84.1; median 56) for adults.

**Figure 1:**
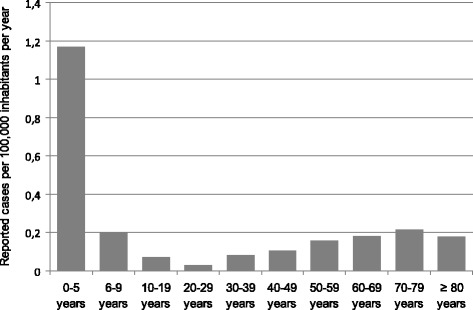
**Age distribution of reported meningitis cases in Germany per 100,000 inhabitants per age group per year from 1992/93 to 2012/13 (n = 3,116) based on a population of 82,437,995 in 2005 (** **http://www.destatis.de** **).**

As expected, children were disproportionately more often affected by meningitis in the first years of life (Figure 1), and the younger the child, the higher the risk: 484 cases occurred within the first year of life (15.7% of all cases), followed by 252 (8.2%), 116 (3.8%), 103 (3.3%), 69 (2.2%) and 44 (1.4%) cases for the next years of age, respectively.

**Figure 2:**
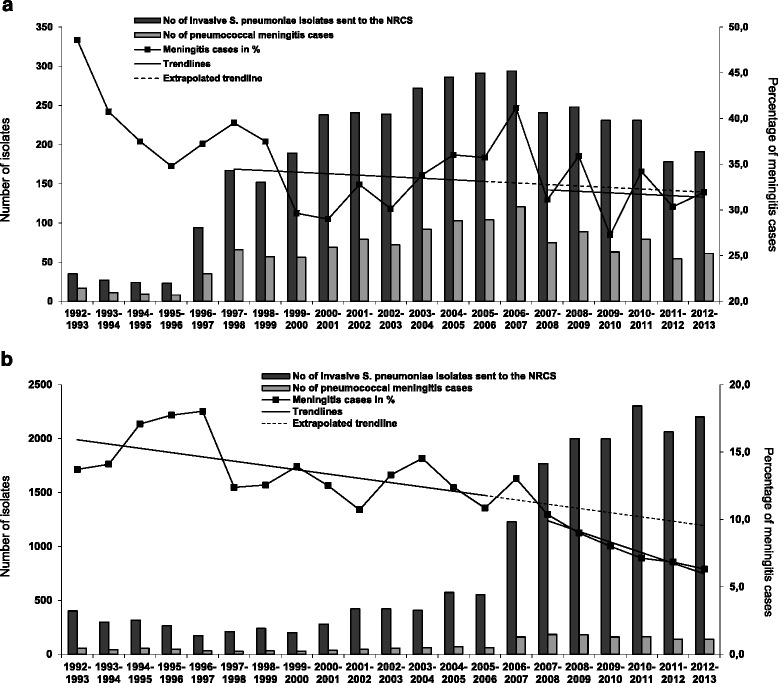
**Number of IPD isolates sent to the NRCS and number of pneumococcal meningitis cases contained. a** Number of IPD isolates from children sent to the NRCS (n = 3,892) and number of pneumococcal meningitis cases contained (n = 1,320) per pneumococcal season from 1992/93 to 2012/13 (left Y-axis). The percentage of meningitis cases is indicated on the right Y-axis and trendlines are added for the pre- (1997/98-2005/06, slope −0.17) and the post-vaccination era (2007/08-2012/13, slope −0.16). The difference between the trendlines was highly significant (p<0.0001). **b** Number of IPD isolates from adults sent to the NRCS (n = 18,312) and number of pneumococcal meningitis cases contained (n = 1,766) per pneumococcal season from 1992/93 to 2012/13 (left Y-axis). The percentage of meningitis cases is indicated on the right Y-axis and trendlines are added for the pre- (1997/98-2005/06, slope −0.32) and the post-vaccination era (2007/08-2012/13, slope −0.79). Both the overall proportion and difference between the trendlines were highly significant (p<0.0001).

After the introduction of conjugate vaccination, the proportion of meningitis cases among all reported 22,204 IPD cases changed for both children and adults. Among children, the percentage of meningitis showed a slightly decreasing trend (slope: −0.17) in the pre-PCV7 period (1997/98-2005/06; the first five seasons were taken out of this analysis because of the low number of reported cases). After vaccine introduction (2007/08-2012/13), a similar decreasing trend (slope: −0.16) was observed. The post-PCV7 trendline shows a significant drop when compared to the projected pre-PCV7 trendline (p<0.0001). Among adults, a decreasing trend in meningitis percentage was found in the pre-PCV7 period as well (1992/93-2005/06, slope: −0.32). Post-vaccination the decrease was also stronger (2007/08-2012/13, slope: −0.79), and the comparison between the projected pre-PCV7 trendline and the post-PCV7 trendline also reached statistical significance (p<0.0001) (Figure 2a and b).

**Figure 3:**
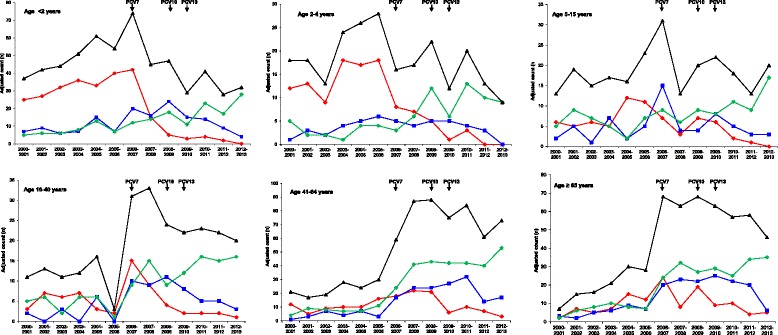
**Isolates from meningitis cases from 2000/01 to 2012/13 (n = 2,508) according to age groups and PCV7 vaccine-type (red), PCV13-non-PCV7 vaccine-type (blue) or non-PCV13 vaccine-type (green).** The number of all meningitis cases is shown in black. The Y-axes of the figure are adjusted to the number of meningitis cases in each age group.

Data on serotypes were available for 3,051 isolates (98.9%), which were included for further analysis. The number of isolates from meningitis cases according to age groups (<2 years, 2–4 years, 5–15 years, 16–40 years, 41–64 years and ≥ 65 years) and PCV7 vaccine-type, PCV13-non-PCV7 vaccine-type (i.e. this includes only the six additional serotypes present in PCV13, as compared to PCV7) and non-PCV13 vaccine-type is shown in Figure 3. Only pneumococcal seasons with a total number of ≥ 100 meningitis cases with information on serotypes available were included in the figure (2000/01-2012/13). A significant decline of PCV7 serotypes (p<0.0001) in the seasons following the introduction of the pneumococcal conjugate vaccines was observed for all children’s and adults’ age groups. The decline continued throughout the higher valent-PCV period (2009/10 -2012/13).

PCV13-non-PCV7 serotypes increased in all age groups after PCV7 introduction (2006/07-2008/09), with age groups <2 years and from 41–64 reaching statistical significance (p<0.0001 and p = 0.0108, respectively). All age groups showed a decrease after the start of higher-valent vaccination (2009/10-2012/13), with age groups <2 years old and 15–40 years old reaching statistical significance (p = 0.0051 and p = 0.0439, respectively).

The serotype distribution among pneumococcal meningitis isolates is shown in Table 1 through Table 2. Overall, the leading serotypes were 14 (9.7%), 7F (7.8%), 3 (6.9%), 19F (5.7%) and 23F (5.0%). Among children, serotypes 14 (16.2%), 7F (8.9%) and 19F (7.1%) were most common, whereas among adults, serotypes 3 (9.6%), 7F (6.9%), 22F (5.0%), 23F (4.9%) and 14 (4.8%) were most prevalent.

**Table 1:** **Serotype distribution of pneumococcal meningitis isolates in Germany among children <16 years, adults ≥16 years, and the overall population (1992/93-2005/06, n = 1382)**

**Serotype**	**Percent of isolates, children**	**Percent of isolates, adults**	**P value**	**Percent of isolates, overall**	**No of isolates, overall**
**14**	23.7	7.9	**<0.0001**	17.5	232
**19F**	9.3	6.0	0.0260	8.2	108
**6B**	9.1	5.1	0.0050	7.7	102
**18C**	8.5	2.8	**<0.0001**	6.3	83
**23F**	6.8	8.9	0.1562	8.1	107
**7F**	6.3	4.6	0.1945	5.8	77
**6A**	4.4	5.5	0.3783	5.1	67
**4**	3.5	7.1	0.0028	5.3	70
**1**	2.8	1.7	0.2063	2.4	32
**19A**	2.4	3.0	0.6153	2.8	37
**3**	1.9	7.1	**<0.0001**	4.4	58
**10A**	1.8	3.6	0.0406	2.7	36
**9V**	1.7	5.6	**0.0001**	3.6	47
**8**	1.3	3.1	0.0221	2.2	29
**33F**	1.0	1.7	0.3452	1.4	18
**11A**	0.9	2.1	0.0686	1.5	20
**15B**	0.9	1.0	1.0000	1.0	13
**9N**	0.8	2.3	0.0220	1.5	20
**22F**	0.6	3.5	**0.0002**	2.0	26
**15C**	0.6	0.3	0.4771	0.5	7
**23A**	0.5	1.7	0.0547	1.1	14
**6C**	0.5	0.5	1.0000	0.5	7
**5**	0.5	0.2	0.3934	0.4	5
**12F**	0.4	1.7	0.0220	1.0	13
**17F**	0.4	1.0	0.1912	0.7	9
**23B**	0.3	0.5	0.6588	0.4	5
**20**	0.3	0.3	1.0000	0.3	4
**2**	0.1	0.3	0.5845	0.2	3
**35B**	0.1	0.0	1.0000	0.1	1
**others**	8.5	10.9	0.1402	10.0	132

Corrected for multiple comparisons using the Dunn-Šidák test (β = 0.001708316).

Significant P values are printed in bold.

**Table 2:** **Serotype distribution of pneumococcal meningitis isolates in Germany among children <16 years, adults ≥16 years, and the overall population (2010/11-2012/13, n = 638)**

**Serotype**	**Percent of isolates, children**	**Percent of isolates, adults**	**P value**	**Percent of isolates, overall**	**No of isolates, overall**
**7F**	8.8	9.1	1.0000	8.8	56
**19A**	7.2	6.6	0.7289	6.6	42
**10A**	7.2	4.0	0.0737	4.9	31
**23B**	7.2	9.6	0.4467	8.6	55
**12F**	6.2	5.9	0.8541	5.8	37
**3**	5.7	9.4	0.2035	8.0	51
**15C**	5.7	2.8	0.1029	3.6	23
**22F**	4.1	5.9	0.5604	5.2	33
**35B**	4.1	1.2	0.0273	2.0	13
**19F**	2.6	3.3	0.8044	3.0	19
**11A**	2.6	4.0	0.4894	3.4	22
**33F**	2.6	1.9	0.5481	2.0	13
**6B**	2.1	0.7	0.2081	1.1	7
**8**	2.1	3.3	0.6054	2.8	18
**15B**	2.1	3.0	0.7897	2.7	17
**9N**	1.5	3.0	0.4139	2.5	16
**6C**	1.5	2.1	1.0000	1.9	12
**23A**	1.5	4.2	0.1462	3.3	21
**18C**	1.0	2.1	0.5180	1.7	11
**23F**	0.5	1.2	0.6731	0.9	6
**1**	0.5	2.1	0.2966	1.6	10
**5**	0.5	0.0	0.3041	0.2	1
**6A**	0.5	1.9	0.2892	1.4	9
**4**	0.0	0.7	0.5571	0.5	3
**9V**	0.0	0.5	1.0000	0.3	2
**14**	0.0	1.9	0.1140	1.3	8
**2**	0.0	0.0	1.0000	0.0	0
**17F**	0.0	0.7	0.5571	0.5	3
**20**	0.0	0.9	0.3197	0.6	4
**others**	22.2	12.2	**0.0010**	14.9	95

Corrected for multiple comparisons using the Dunn-Šidák test (β = 0.001708316).

Significant P values are printed in bold.

When considering only the pre-PCV7 period (Table 1), the most common serotypes in children were 14 (23.7%), 19F (9.3%), 6B (9.1%), 18C (8.5%), and 23F (6.8%); for adults, the most common pre-PCV7 serotypes were 23F (8.9%), 14 (7.9%), 4 (7.1%), 3 (7.1%), and 19F (6.0%). Of the most prevalent serotypes before vaccination, 14 (23.7% in children vs. 7.9% in adults, p<0.0001), and 18C (8.5% vs. 2.8%, p<0.0001) were significantly more common in children (age <16 years). Among adults, serotypes 3 (7.1% vs. 1.9%, p<0.0001), 9V (5.6% vs. 1.7%, p = 0.0001) and 22F (3.5% vs 0.6%, p = 0.0002), were significantly more prevalent.

The post-PCV7 period (Table 3) already showed a shift in serotype distribution with serotypes 7F (16.7%), 18C (7.9%), 1 (7.0%), 3 (4.8%), 6A (4.8%), and 14 (4.8%) becoming dominant in children, while 3 (12.8%), 7F (6.9%), 22F (6.3%), 6A (5.4%), 10A (5.0%) and 19A (5.0%) led among adult meningitis cases. In the post-PCV7 period, serotypes 7F (16.7% vs 6.9%, p = 0.0001) and 18C (7.9% vs 2.5%, p = 0.0011) were significantly more common in children than in adults, and serotype 3 remained significantly more common in adults (12.8% vs 4.8%, p = 0.0007).

**Table 3:** **Serotype distribution of pneumococcal meningitis isolates in Germany among children <16 years, adults ≥16 years, and the overall population (2007/08-2009/10, n = 750)**

**Serotype**	**Percent of isolates, children**	**Percent of isolates, adults**	**P value**	**Percent of isolates, overall**	**No of isolates, overall**
**7F**	16.7	6.9	**0.0001**	9.6	74
**18C**	7.9	2.5	**0.0011**	4.0	31
**1**	7.0	2.9	0.0149	4.0	31
**14**	4.8	2.7	0.1817	3.3	25
**3**	4.8	12.8	**0.0007**	10.2	78
**6A**	4.8	5.4	0.8593	5.1	39
**6B**	4.4	1.1	0.0100	2.1	16
**22F**	4.4	6.3	0.3927	5.6	43
**19A**	4.0	5.0	0.7066	4.6	35
**15C**	4.0	1.3	0.0288	2.1	16
**10A**	3.5	5.0	0.4487	4.4	34
**19F**	2.2	4.8	0.1081	3.9	30
**23F**	2.2	3.4	0.4908	3.0	23
**8**	2.2	2.7	0.8052	2.5	19
**12F**	2.2	2.7	0.8052	2.5	19
**15B**	1.8	3.1	0.4595	2.6	20
**23A**	1.8	2.9	0.4569	2.5	19
**9N**	1.3	2.9	0.2992	2.3	18
**17F**	1.3	0.6	0.3740	0.8	6
**33F**	1.3	1.9	0.7640	1.7	13
**9V**	0.9	2.3	0.2484	1.8	14
**6C**	0.9	2.9	0.1126	2.2	17
**35B**	0.9	1.3	0.7305	1.2	9
**4**	0.4	2.3	0.1232	1.7	13
**11A**	0.4	2.1	0.1196	1.6	12
**23B**	0.4	2.1	0.1196	1.6	12
**5**	0.0	0.2	1.0000	0.1	1
**2**	0.0	0.0	1.0000	0.0	0
**20**	0.0	0.6	0.5575	0.4	3
**others**	13.2	9.6	0.1564	10.4	80

Corrected for multiple comparisons using the Dunn-Šidák test (β = 0.001708316).

Significant P values are printed in bold.

The post-PCV13 period (Table 2), which includes the higher-valent formulations of PCV, shows further changes to the serotype distribution in meningitis cases. Leading serotypes from children in this period were 7F (8.8%), 19A (7.2%), 10A (7.2%), 23B (7.2%), and 12F (6.2%); in adults, 23B (9.6%), 3 (9.4%), 7F (9.1%), 19A (6.6%), 22F (5.9%) and 12F (5.9%) occurred most often. In the post-PCV13 period, there were no serotypes that were significantly more prevalent in either children or adults.

When comparing the serotype distribution in the pre PCV7-period (1992/93-2005/06), the post PCV7-period (2007/08-2009/10) and the post PCV13-period (2010/11-2012/13), several changes were observed. Among children (Table 4), serotypes 4, 6B, 14, 19F and 23F decreased significantly when comparing the pre- and post- vaccination periods (pre-PCV7 vs. post-PCV7 + post-PCV13, p-values less than β = 0.000732493). Among the six additional serotypes included in PCV13, only serotype 6A showed a decrease post vaccination, although it does not reach statistical significance. The comparison of the pre-PCV7 with the post-PCV7 period, however, shows that serotypes 1, 3, 6A, 7F and 19A all increased in the post-PCV7 period, with only serotype 7F reaching statistical significance, according to our very stringent criteria. However, comparing the post-PCV7 period with the post-PCV13 period showed a decrease in serotypes 1, 6A and 7F, with only serotype 1 reaching statistical significance. Non-PCV13 serotypes which increased significantly after the introduction of conjugate vaccines were 12F, 15C, 22F, 23B and 35B.

**Table 4:** **Most common serotypes among pneumococcal meningitis isolates in Germany among children <16 years (1992/93-2005/06, n = 777; 2007/08-2009/10, n = 227; 2010/11-2012/13, n = 194)**

**Children**	**1992/93-2005/06**		**2007/08-2009/10**		**2010/11-2012/13**		**P values**		
**Serotypes**	**(n)**	**%**	**(n)**	**%**	**(n)**	**%**	**Pre PCV 7 vs post PCV7+13**	**Pre PCV7 vs post PCV7**	**Post PCV7 vs post PCV13**
**4**	27	3.5	1	0.4	0	0.0	**0.0002↓**	0.0105**↓**	1.0000
**6B**	71	9.1	10	4.4	4	2.1	**0.0001↓**	0.0188**↓**	0.2755**↓**
**9V**	13	1.7	2	0.9	0	0.0	0.1015**↓**	0.5414**↓**	0.5019**↓**
**14**	184	23.7	11	4.8	0	0.0	**<0.0001↓**	**<0.0001↓**	0.0012**↓**
**18C**	66	8.5	18	7.9	2	1.0	0.0186**↓**	0.8918**↓**	0.0008**↓**
**19F**	72	9.3	5	2.2	5	2.6	**<0.0001↓**	**0.0002↓**	1.0000
**23F**	53	6.8	5	2.2	1	0.5	**<0.0001↓**	0.0088**↓**	0.2241**↓**
**PCV 7**	486	62.5	52	22.9	12	6.2	**<0.0001↓**	**<0.0001↓**	**<0.0001↓**
**1**	22	2.8	16	7.0	1	0.5	0.3062**↑**	0.0087**↑**	**0.0007↓**
**5**	4	0.5	0	0.0	1	0.5	0.6624**↓**	0.5799**↓**	0.4608**↑**
**7F**	49	6.3	38	16.7	17	8.8	**0.0001↑**	**<0.0001↑**	0.0197**↓**
**PCV10**	561	72.2	106	46.7	31	16.0	**<0.0001↓**	**<0.0001↓**	**<0.0001↓**
**3**	15	1.9	11	4.8	11	5.7	0.0025**↑**	0.0287**↑**	0.8269**↑**
**6A**	34	4.4	11	4.8	1	0.5	0.2106**↑**	0.7184**↑**	0.0074**↓**
**19A**	19	2.4	9	4.0	14	7.2	0.0084**↑**	0.2504**↑**	0.1961**↑**
**PCV13**	629	81.0	137	60.4	57	29.4	**<0.0001↓**	**<0.0001↓**	**<0.0001↓**
**2**	1	0.1	0	0.0	0	0.0	1.0000	1.0000	1.0000
**8**	10	1.3	5	2.2	4	2.1	0.3323**↑**	0.3497**↑**	1.0000
**9N**	6	0.8	3	1.3	3	1.5	0.3624**↑**	0.4309**↑**	1.0000
**10A**	14	1.8	8	3.5	14	7.2	0.0021**↑**	0.1255**↑**	0.1230**↑**
**11A**	7	0.9	1	0.4	5	2.6	0.3969**↑**	0.6912**↓**	0.0991**↑**
**12F**	3	0.4	5	2.2	12	6.2	**<0.0001↑**	0.0174**↑**	0.0472**↑**
**15B**	7	0.9	4	1.8	4	2.1	0.1734**↑**	0.2811**↑**	1.0000
**17F**	3	0.4	3	1.3	0	0.0	0.4295**↑**	0.1329**↑**	0.2528**↓**
**20**	2	0.3	0	0.0	0	0.0	0.5438**↓**	1.0000	1.0000
**22F**	5	0.6	10	4.4	8	4.1	**<0.0001↑**	**0.0003↑**	1.0000
**33F**	8	1.0	3	1.3	5	2.6	0.2906**↑**	0.7189**↑**	0.4793**↑**
**PPV23**	729	93.8	199	87.7	137	70.6	**<0.0001↓**	0.6504**↓**	**<0.0001↓**
**6C**	4	0.5	2	0.9	3	1.5	0.2917**↑**	0.6226**↑**	0.6653**↑**
**15C**	5	0.6	9	4.0	11	5.7	**<0.0001↑**	0.0009**↑**	0.4930**↑**
**23A**	4	0.5	4	1.8	3	1.5	0.0588**↑**	0.0826**↑**	1.0000
**23B**	2	0.3	1	0.4	14	7.2	**<0.0001↑**	0.5369**↑**	**0.0002↑**
**35B**	1	0.1	2	0.9	8	4.1	**0.0002↑**	0.1300**↑**	0.0492**↑**
**others**	66	8.5	30	13.2	43	22.2	**<0.0001↑**	0.0388**↑**	0.0201**↑**
**NVT**	82	10.6	48	21.1	82	42.3	**<0.0001↑**	**0.0001↑**	**<0.0001↑**

Corrected for multiple comparisons using the Dunn-Šidák test (β = 0.000732493). Arrows indicate increasing or decreasing percentage of serotypes post vaccination.

PCV7: serotypes 4, 6B, 9V, 14, 18C, 19F, 23F; PCV10: PCV7 + serotypes 1, 5, 7F; PCV13: PCV10 + serotypes 3, 6A, 19A; PPV23: PCV13 + serotypes 2, 8, 9N, 10A, 11A, 12F, 15B, 17F, 20, 22F, 33F, but without serotyp 6A; NVT: non-vaccine types, i.e. serotypes not included in any of the above mentioned vaccines.

Significant P values are printed in bold.

Among adults (Table 5), a significant decrease of serotypes 4, 6B, 9V, 14, and 23F was observed after the start of childhood conjugate vaccination. Serotypes 1, 3, 7F and 19A increased post-PCV7 as compared to pre-PCV7, but not significantly. When comparing the post-PCV13 period to the post-PCV7 period, serotypes 1, 3 and 6A appear to decrease again, with serotype 6A approaching statistical significance. Serotypes 7F and 19A still showed an increase post-PCV13, but less pronounced (higher p-values). Non-PCV13 serotypes which increased among adults following the introduction of childhood-vaccination were 6C, 12F, 15B, 15C, 22F, 23A, 23B and 35B. Only the increase of serotype 23B reached statistical significance (p<0.0001).

**Table 5:** **Most common serotypes among pneumococcal meningitis isolates in Germany among adults ≥16 years (1992/93-2005/06, n = 605; 2007/08-2009/10, n = 523; 2010/11-2012/13, n = 444)**

**Adults**	**1992/93-2005/06**		**2007/08-2009/10**		**2010/11-2012/13**		**P values**		
**Serotypes**	**(n)**	**%**	**(n)**	**%**	**(n)**	**%**	**pre PCV7 vs post PCV7+13**	**pre PCV7 vs post PCV7**	**post PCV7 vs post PCV13**
**4**	43	7.1	12	2.3	3	0.7	**<0.0001↓**	**0.0001↓**	0.0644**↓**
**6B**	31	5.1	6	1.1	3	0.7	**<0.0001↓**	**0.0002↓**	0.5189**↓**
**9V**	34	5.6	12	2.3	2	0.5	**<0.0001↓**	0.0060**↓**	0.0270**↓**
**14**	48	7.9	14	2.7	8	1.9	**<0.0001↓**	0.0001**↓**	0.3952**↓**
**18C**	17	2.8	13	2.5	9	2.1	0.5094**↓**	0.8533**↓**	0.6715**↓**
**19F**	36	6.0	25	4.8	14	3.3	0.0892**↓**	0.4295**↓**	0.2510**↓**
**23F**	54	8.9	18	3.4	5	1.2	**<0.0001↓**	**0.0001↓**	0.0198**↓**
**PCV 7**	263	43.5	100	19.1	44	9.9	**<0.0001↓**	**<0.0001↓**	**0.0001↓**
**1**	10	1.7	15	2.9	9	2.1	0.2918**↑**	0.2232**↑**	0.5347**↓**
**5**	1	0.2	1	0.2	0	0.0	1.0000	1.0000	1.0000
**7F**	28	4.6	36	6.9	39	9.1	0.0158**↑**	0.1212**↑**	0.2799**↑**
**PCV10**	302	49.9	152	29.1	92	9.4	**<0.0001↓**	**<0.0001↓**	0.0030**↓**
**3**	43	7.1	67	12.8	40	9.4	0.0103**↑**	0.0017**↑**	0.0645**↓**
**6A**	33	5.5	28	5.4	8	1.9	0.1283**↓**	1.0000	0.0035**↓**
**19A**	18	3.0	26	5.0	28	6.6	0.0180**↑**	0.0912**↑**	0.4007**↑**
**PCV13**	396	65.5	273	52.2	168	17.2	**<0.0001↓**	**<0.0001↓**	**<0.0001↓**
**2**	2	0.3	0	0.0	0	0.0	0.1480**↓**	0.5022**↓**	1.0000
**8**	19	3.1	14	2.7	14	3.3	0.7636**↓**	0.7246**↓**	0.7032**↑**
**9N**	14	2.3	15	2.9	13	3.0	0.5245**↑**	0.5768**↑**	1.0000
**10A**	22	3.6	26	5.0	17	4.0	0.5154**↑**	0.3016**↑**	0.4360**↓**
**11A**	13	2.1	11	2.1	17	4.0	0.4186**↓**	1.0000	0.1256**↑**
**12F**	10	1.7	14	2.7	25	5.9	0.0072**↑**	0.3013**↑**	0.0219**↑**
**15B**	6	1.0	16	3.1	13	3.0	0.0080**↑**	0.0161**↑**	1.0000
**17F**	6	1.0	3	0.6	3	0.7	0.5530**↓**	0.5165**↓**	1.0000
**20**	2	0.3	3	0.6	4	0.9	0.4958**↑**	0.6677**↑**	0.7091**↑**
**22F**	21	3.5	33	6.3	25	5.9	0.0321**↑**	0.0351**↑**	0.6856**↓**
**33F**	10	1.7	10	1.9	8	1.9	0.8463**↑**	0.8227**↑**	1.0000
**PPV23**	488	80.7	511	97.7	375	68	**<0.0001↑**	0.0146**↑**	0.0153**↓**
**6C**	3	0.5	15	2.9	9	2.1	0.0023**↑**	0.0016**↑**	0.5347**↓**
**15C**	2	0.3	7	1.3	12	2.8	0.0055**↑**	0.0900**↑**	0.1634**↑**
**23A**	10	1.7	15	2.9	18	4.2	0.0392**↑**	0.2232**↑**	0.3749**↑**
**23B**	3	0.5	11	2.1	41	9.6	**<0.0001↑**	0.0272**↑**	**<0.0001**
**35B**	0	0.0	7	1.3	5	1.2	0.0048**↑**	0.0045**↑**	1.0000
**others**	66	10.9	50	9.6	52	12.2	0.8668**↓**	0.4921**↓**	0.2945**↑**
**NVT**	84	13.9	105	20.1	137	32.1	**<0.0001↑**	0.0065**↑**	**0.0001↑**

Corrected for multiple comparisons using the Dunn-Šidák test (β = 0.000732493). Arrows indicate increasing or decreasing percentage of serotypes post vaccination.

PCV7: serotypes 4, 6B, 9V, 14, 18C, 19F, 23F; PCV10: PCV7 + serotypes 1, 5, 7F; PCV13: PCV10 + serotypes 3, 6A, 19A; PPV23: PCV13 + serotypes 2, 8, 9N, 10A, 11A, 12F, 15B, 17F, 20, 22F, 33F, but without serotyp 6A; NVT: non-vaccine types, i.e. serotypes not included in any of the above mentioned vaccines.

Significant P values are printed in bold.

In the pre-PCV7 era, theoretical serotype coverage of PCV7 for children was 62.5%, and for PCV10 and PCV13, the serotype coverage was 72.2% and 81.0%, respectively. The theoretical coverage of PPV23 was 85.1%. Among adults, coverage for the 7-, 10-, 13- and 23-valent vaccines was 43.5%, 49.9%, 65.5% and 80.7%, respectively. PCV13-non-PCV7 serotypes accounted for 18.4% and 22.0% of all serotypes in children and adults respectively. For all vaccines types, the coverage was higher among children than among adults (Table 6).

**Table 6:** **Serotype coverage (%) of the 7-, 10-, and 13-valent pneumococcal conjugate vaccines (7v, 10v, 13v), the 23-valent pneumococcal polysaccharide vaccine (23v), and of the serotypes included in the 13- but not in the 7-valent pneumococcal conjugate vaccine (13vnon7v) among children <16 years and adults ≥16 years (1992/93-2005/06, n = 1, 382; 2007/08-2009/10, n = 750; 2010/11-2012/13, n = 638)**

	**Children <16 years**					**Adults ≥16 years**				
**Year**	**7v**	**10v**	**13v**	**13v non 7v**	**23v**	**7v**	**10v**	**13v**	**13v non 7v**	**23v**
1992-1993	52.9	52.9	82.4	29.4	64.7	43.8	56.3	64.6	20.8	81.3
1993-1994	54.5	54.5	63.6	9.1	90.9	31.0	35.7	52.4	21.4	69.0
1994-1995	88.9	88.9	100.0	11.1	100.0	31.3	39.6	60.4	29.2	79.2
1995-1996	75.0	75.0	75.0	0.0	87.5	41.9	51.2	67.4	25.6	93.0
1996-1997	41.2	64.7	79.4	38.2	73.5	56.0	60.0	68.0	12.0	76.0
1997-1998	60.6	66.7	72.7	12.1	77.3	45.0	50.0	75.0	30.0	75.0
1998-1999	64.9	73.7	84.2	19.3	93.0	48.0	52.0	72.0	24.0	80.0
1999-2000	75.0	83.9	87.5	12.5	89.3	57.1	64.3	78.6	21.4	96.4
2000-2001	62.3	71.0	78.3	15.9	81.2	51.4	57.1	68.6	17.1	85.7
2001-2002	57.0	68.4	78.5	21.5	89.9	42.2	42.2	53.3	11.1	73.3
2002-2003	65.3	75.0	79.2	13.9	86.1	41.1	50.0	67.9	26.8	76.8
2003-2004	64.1	73.9	84.8	20.7	84.8	44.1	49.2	61.0	16.9	78.0
2004-2005	60.2	68.0	81.6	21.4	82.5	39.4	50.7	70.4	31.0	78.9
2005-2006	65.4	78.8	82.7	17.3	89.4	51.7	51.7	68.3	16.7	88.3
1992/93-2005/06	62.5	72.2	81.0	18.4	85.1	43.5	49.9	65.5	22.0	80.7
(pre PCV7)										
2006-2007	47.1	63.6	80.2	33.1	85.1	35.0	46.9	64.4	29.4	80.6
2007-2008	33.3	52.0	65.3	32.0	76.0	21.3	34.4	51.9	30.6	79.2
2008-2009	19.1	43.8	56.2	37.1	73.0	24.4	32.2	56.1	31.7	76.1
2009-2010	15.9	44.4	60.3	44.4	73.0	10.6	19.4	48.1	37.5	67.5
2007/08-2009/10	22.9	46.7	60.4	37.4	74.0	19.1	29.1	52.2	33.1	74.6
(post PCV7)										
2010-2011	11.4	21.5	40.5	29.1	55.7	13.4	25.6	49.4	36.0	74.4
2011-2012	5.6	24.1	33.3	27.8	64.8	9.2	22.0	36.9	27.7	67.4
2012-2013	0.0	1.6	11.5	11.5	52.5	6.5	13.7	25.2	18.7	59.0
2010/11-2012/13	6.2	16.0	29.4	23.2	57.2	9.9	20.7	37.8	27.9	67.3
(post PCV13)										
pre7 vs. post7+post13	<0.0001	<0.0001	<0.0001	0.0137	0.0004	<0.0001	<0.0001	0.0001	0.0140	0.0130
pre7 vs. post7	<0.0001	0.0010	0.0010	0.0024	0.0539	0.0001	0.0005	0.0065	0.0096	0.1900
post7 vs. post13	0.0529	0.0148	0.0243	0.0887	0.0129	0.1219	0.2328	0.1130	0.3448	0.2637

Corrected for multiple comparisons using the Dunn-Šidák test (β = 0. 001708316).

During the post PCV7- and post-PCV13 period, the vaccine coverage for the 7-, 10-, 13- and 23-valent vaccines decreased to 0.0%, 1.6%, 11.5% and 52.5% for children, and 6.5%, 13.7%, 25.2% and 59.0% for adults in 2012/13 (PCV13-non-PCV7 serotypes decreased to 11.5% and 18.7% respectively). The decrease in coverage during the post-vaccination period was statistically significant for all three PCVs both for children and adults. For PCV13-non-PCV7 serotypes, there was an increase in the post-PCV7 period when compared to the pre-PCV7 period (reaching statistical significance for children, and near statistical significance for adults), followed by a decrease in children and adults in the post-PCV13 period. Since cross reactive serotypes were not included in the calculation, coverage information strictly refers to the serotypes included in the vaccines.

Detailed results of the antimicrobial susceptibility testing are shown in Table 7. Generally, the differences in antibiotic susceptibility between children < 16 years and adults ≥ 16 years were low. Macrolides showed a significant difference when comparing rates of resistance in children (19.8%) against rates of resistance in adults (10.8%; p < 0.0001), as well as a significant change when comparing the pre- and post-vaccination periods in children (25.1% vs. 9.7%; p<0.0001). Following vaccine introduction, the difference between resistant isolates in children and adults diminished and lost statistical significance (post-PCV7: p = 0.6820, post PCV13: p = 0.8803), due to decreasing resistance rates in the post-vaccination period in both age groups.

**Table 7:** **Antibiotic susceptibilities of pneumococcal meningitis isolates among children <16 years and adults ≥16 years in Germany from 1992/93 to 2012/13**

	**Children <16 years**			**Adults ≥16 years**			**CLSI breakpoints (mg/L)**	
	**I%**	**R%**	**n**	**I%**	**R%**	**n**	**I**	**R**
Penicillin G, meningitis	0.0	7.2	1,317	0.0	7.2	1,731	-	≥0.12
Cefotaxime, meningitis	0.8	0.5	1,317	0.9	0.2	1,729	1	≥2
Macrolides*	0.1	19.8	1,317	0.1	10.8	1,730	0.5	≥1
Clindamycin	0.2	5.9	1,316	0.5	5.7	1,730	0.5	≥1
Tetracycline	0.4	9.2	1,316	0.9	8.9	1,731	2	≥4
Levofloxacin	0.2	0.3	874	0.2	0.1	1,326	4	≥8
SXT	6.1	5.7	870	7.1	5.5	1,318	1/19-2/38	≥4/76

I, intermediately-resistant; R, resistant; n, number of isolates. SXT, trimethoprim-sulfamethoxazole. MIC breakpoints according to CLSI M100-S24, 2014 [20].

*Macrolides: erythromycin or clarithromycin.

## Discussion

Here we present the results of 20 years of pneumococcal meningitis surveillance in Germany.

Some limitations must be considered when interpreting the results of this study. First, the isolates were sent by the participating laboratories on a voluntary basis, as IPD is not a notifiable disease in Germany which makes the system more vulnerable to under-reporting. Certainly, this would affect non-meningitis IPD more than meningitis, since the latter is a more severe condition. Furthermore, the systematic sampling of invasive isolates from adults (1992) and children (1997) was taken up at different times, and included population-based studies in three German federal states: North Rhine-Westphalia, started in 2001; Bavaria, started in 2006; Saxony, started in 2006. Moreover, the structure of the surveillance project has been continuously improved over the 20 years of the study period, particularly after the general recommendation of pneumococcal conjugate vaccination [21], which led to increased disease awareness by both clinical microbiologists and pediatricians. The introduction of a web-based reporting system, Pneumoweb, in 2007 also contributed to an increased number of reported cases of IPD among adults, from 200–500 cases per season to over 2000 cases per season.

As expected [22], we observed a peak incidence of pneumococcal meningitis in patients younger than two years. For both children and adults, the frequency of meningitis, measured as a fraction of total IPD, has been decreasing since the start of our surveillance. The fact that this decrease became stronger post PCV7-vaccination may indicate a beneficial effect of vaccination on the severity of pneumococcal disease: apparently, the serotypes remaining after vaccination are less capable of causing meningitis. The general decrease in frequency of pneumococcal meningitis among children since the general recommendation for PCV vaccination has been described by our group previously [23]. The here-reported significant decrease of meningitis caused by PCV7 and PCV13 serotypes post-vaccination in children <2 years of age shows the effectiveness of these vaccinations in this age group. As we observed a decrease in PCV7 and PCV13 serotypes among older children and adults as well, our data suggest a strong herd protection effect.

Our data are in line with previous observations [2,24,25] and underline the efficacy of vaccination strategies to strengthen herd protection [26]. The apparent increase of meningitis cases among adults since 2006/07 is most likely due to enhanced surveillance, as described in our study limitations above.

According to our study, in the pre-PCV7 period, serotype 14 was the most prevalent serotype among meningitis cases, followed in frequency by the serotypes 19F, 23F, 6B and 18C. Serotype 14 has been described to be the most frequent serotype in meningitis in Italy, Belgium and Brazil, and in Germany, among both children and adults [27-31]. Serogroups 6 and 23 have been previously described to be among the major players in pneumococcal meningitis [6,27-29]. In the post-PCV7- and post-PCV13 periods, the serotype distribution drastically changed, with the vaccine serotypes loosing importance.

In the pre-PCV7 period, serotypes significantly more common in children in our study were serotypes 14 and 18C. Serotype 14 is among the serotypes often defined as ‘pediatric serotypes’ (6B, 9V, 14, 19F, and 23F) [32], and serotype 18C is among the ten serotypes reported to be responsible for about 80% of IPD in children younger than 5 years [33]. Four of these six serotypes were among the most common in IPD among German children <16 years in the pre-vaccination period (serotype 14, 26.4%; 6B, 7.7%; 19F, 7.1%, 18C, 6.2%), as recently published by our group [30]. In the post-PCV7 period 7F appeared as significantly more prevalent serotype among children. In the post-PCV13 period there were no serotypes that were significantly more prevalent in either children or adults.

In the pre-vaccination period, among adults, serotypes 23F, 14, 3 and 4 were predominant, three of which were among the more common in IPD among adults ≥16 years in Germany (serotype 14, 14.3%; 3, 9.2%; 23F, 5.6%) [31]. Also here, the serotype distribution drastically changed in the post-PCV7- and post-PCV13 periods, with the vaccine serotypes loosing importance. In the post-PCV7 period only serotype 3 remained significantly more prevalent among adults.

In the pre-PCV7 period, serotypes 3, 9V and 22F were significantly more common in adults than in children and can be considered ‘adult serotypes’.

A reduction in PCV7 vaccine serotypes was observed post-PCV7-vaccination in both children and adults but did not reach statistical significance in either category for every single PCV7 serotype. Among children, serotypes 9V (p = 0.1015) and 18C (p = 0.0186) both decreased, but the p-values did not meet our very stringent criteria. Among adults, the same was true for 19F (p = 0.0892) whereas 18C showed no significant change at all (p = 0.5094). This could indicate a reservoir for this serotype among adults. The increase of serotypes included in the higher-valent pneumococcal conjugate vaccines in both children and adults in the post-PCV7 period shows the rationale for introduction of these vaccines in Germany (PCV10: April 2009, PCV13 December 2009). In the post-PCV13 period, a decrease in serotypes 1, 6A and 7F in children, and in serotypes 1, 3 and 6A in adults was observed. Additionally, the observed increase in serotype 19A in the post-PCV7 period was much less pronounced in the post-PCV13 period. These shifts in serotype prevalence show that the increase in non-vaccine serotypes after introduction of PCV7 in childhood vaccination was, for a large part, caused by the serotypes that are included in PCV13, and thus, that the introduction of PCV13 has reversed these trends.

However, there is an increase of serotypes not included in any conjugate vaccine (and many serotypes not in the polysaccharide vaccine). As discussed before [24], non-vaccine types have emerged after 2006. This pattern has been recently reported in the northern region of France, where introduction of the PCV7 vaccine showed success in the beginning, but was followed by a rebound of meningitis incidence to the pre-vaccination era [34]. As a substantial proportion of cases were due to serotypes covered by the PCV13 vaccine, the authors recommended the near-term introduction of higher-valent vaccines. Previous studies in children indicate that pneumococcal meningitis caused by non-PCV7 serotypes was associated with a lower lethality than PCV7 serotypes, while the incidence of neurological deficits was the same in both groups [35]. Moreover, Nigrovic and colleagues confirmed that *S. pneumoniae* is still the responsible pathogen in a substantial proportion of patients with bacterial meningitis in childhood, with half of them caused by non-PCV7-vaccine serotypes [36]. Obviously, serotypes not covered by pneumococcal conjugate vaccines have to be regarded as a persisting clinical threat, requiring increased efforts for the implementation of vaccines with broader coverage.

Comparing the theoretical serotype coverage for the pneumococcal vaccines among meningitis cases, and for all IPD cases in the era before pneumococcal conjugate vaccination, the coverage was similar both for children (7v, 62.5% vs. 62.3%; 10v, 72.2% vs. 75.5%; 13v, 81.0% vs. 84.8%; 23v, 85.1% vs. 87.3%) and adults (7v, 43.5% vs. 46.5%; 10v, 49.9% vs. 61.1%; 13v, 65.5% vs. 76.3%; 23v, 80.7% vs. 88.7%) [30]. The significant decrease in serotype coverage for PCV7 and PCV13 post vaccination shows the impact of the childhood vaccination program on meningitis among children, as well as the resulting herd protection effect on meningitis among adults [21]. These findings are in line with the literature, as a decline in coverage following the introduction of pneumococcal conjugate vaccination has been described in other countries as well [2,24].

In general, the prevalence of antibiotic-resistant pneumococcal isolates continues to increase worldwide [37,38], with relevant differences between countries and continents [39,40]. According to the current CLSI guidelines [20], different susceptibility breakpoints have to be applied for penicillin G with regard to meningitis and non-meningitis cases. All isolates associated with meningitis that were formerly categorized as intermediate are now regarded as resistant, which results in higher resistance rates and should be kept in mind when interpreting surveillance studies on pneumococcal penicillin resistance [41,42]. The difference concerning the macrolide nonsusceptibility among children and adults in our study can be attributed to the higher resistance rates among children observed in the years from 2000 to 2007 [43]. The introduction of childhood vaccination has reduced these rates significantly (p<0.0001), since most resistant isolates were found among vaccine serotypes. With regard to findings in other countries, the antibiotic sensitivity of *S. pneumoniae* isolates detected in Germany is still relatively low [4,6,24,27-29,44].

Based on serotype distribution, antimicrobial susceptibility and theoretical vaccine coverage among pneumococcal meningitis cases in Germany from 1992/93 to 2012/13, our study shows the impact of PCV7 and PCV13 on clinical practice. These findings have to be considered in the context of the serious burden of disease as *Streptococcus pneumoniae* represents one of the most frequent pathogens in acute bacterial meningitis, often leading to sustained neurological deficits in survivors. In light of the relevance of the clonal and capsular types for the clinical pathogenicity of pneumococci [45], our data suggest that the introduction of PCV7 and PCV13 in the German pediatric immunization program has had an important influence on the spectrum of pneumococcal meningitis across all ages. It remains to be clarified whether such differences, and the impact of vaccination programs, may also contribute to the heterogeneity of therapeutic responses in acute bacterial meningitis.

## Conclusions

The serotype distributions among both children and adults showed drastic changes, with the vaccine-serotypes loosing importance. The reduction in prevalence of vaccine-serotypes among adults clearly suggests a herd-protection effect. Changes in serotype prevalence must be continuously monitored to observe future trends concerning pneumococcal meningitis.
